# A Novel Biomarker Panel to Identify Steroid Resistance in Childhood Idiopathic Nephrotic Syndrome

**DOI:** 10.1177/1177271917695832

**Published:** 2017-03-08

**Authors:** Michael R Bennett, LaTawnya Pleasant, Christopher Haffner, Qing Ma, Wendy D Haffey, Jun Ying, Michael Wagner, Kenneth D Greis, Prasad Devarajan

**Affiliations:** 1 Division of Nephrology and Hypertension, Cincinnati Children’s Hospital Medical Center, Cincinnati, OH, USA; 2 Division of Biomedical Informatics, Cincinnati Children’s Hospital Medical Center, Cincinnati, OH, USA; 3 Department of Cancer and Cell Biology, University of Cincinnati College of Medicine, Cincinnati, OH, USA; 4 Department of Environmental Health, University of Cincinnati College of Medicine, Cincinnati, OH, USA

**Keywords:** Nephrotic syndrome, steroid resistance, focal segmental glomerulosclerosis, minimal change disease, biomarkers

## Abstract

Idiopathic nephrotic syndrome (NS) is the most common glomerular disorder of childhood. Response to initial treatment with corticosteroids is an indicator of prognosis, as resistant patients often present more progressive disease. In this cross-sectional pilot study, we set out to discover a panel of noninvasive biomarkers that could distinguish steroid-resistant nephrotic syndrome (SRNS) from steroid-sensitive nephrotic syndrome (SSNS). Information gleaned from such a panel could yield more individualized treatment plans and prevent unnecessary steroid exposure in patients unlikely to respond. Urine was collected from 50 pediatric patients diagnosed with idiopathic NS at Cincinnati Children’s Hospital Medical Center. Isobaric tags for relative and absolute quantitation (iTRAQ) was used to discover 13 proteins that were differentially expressed in SSNS vs SRNS in a small 5 × 5 discovery cohort. Suitable assays were found for 9 of the 13 markers identified by iTRAQ and were used in a 25 SRNS × 25 SSNS validation cohort. Vitamin D–binding protein (VDBP), alpha-1 acid glycoprotein 1 (AGP1), alpha-1 acid glycoprotein 2 (AGP2), alpha-1-B glycoprotein (A1BG), fetuin-A, prealbumin, thyroxine-binding globulin and hemopexin, and alpha-2 macroglobulin were measured and combined with urine neutrophil gelatinase–associated lipocalin (NGAL), which had been previously shown to distinguish patients with SRNS. Urinary VDBP, prealbumin, NGAL, fetuin-A, and AGP2 were found to be significantly elevated in SRNS using univariate analysis, with area under the receiver operating characteristic curves (AUCs) ranging from 0.65 to 0.81. Multivariate analysis revealed a panel of all 10 markers that yielded an AUC of 0.92 for identification of SRNS. A subset of 5 markers (including VDBP, NGAL, fetuin-A, prealbumin, and AGP2) showed significant associations with SRNS and yielded an AUC of 0.85.

## Introduction

Idiopathic nephrotic syndrome (NS) is the leading glomerular disease in pediatric patients, occurring in 16 per 100 000 children.^1^ Initial presentation of various NS subtypes is similar and includes the presence of proteinuria, edema, hypoalbuminemia, and hypercholesterolemia. Despite initial similarities, NS subtypes have markedly different disease courses and outcomes. Invasive biopsy remains the only method for positive diagnosis, and the 2 most frequent histopathological findings are focal segmental glomerulosclerosis (FSGS) and minimal change disease (MCD). Prognosis depends on underlying pathophysiology and response to steroid treatment. Approximately 95% of children with MCD achieve remission following an 8-week course of prednisone (steroid-sensitive nephrotic syndrome [SSNS]) compared with 80% of patients with FSGS who fail to reach remission in response to steroids (steroid-resistant nephrotic syndrome [SRNS]).^2^ Focal segmental glomerulosclerosis is the most common acquired cause of end-stage renal disease in children and leads to further complications with roughly 30% recurrence posttransplant.^3,4^

Although kidney biopsies are effective for diagnosis in the adult population, they are not typically performed at presentation in children because therapeutic response better predicts long-term outcomes than histology in the pediatric population, and FSGS is often underdiagnosed due to its focal nature in combination with a smaller core size.^2,5^ As a result, response to treatment is used as a one-size-fits-most diagnostic tool. The problem with this approach is that a population of children who are unlikely to respond to steroids (FSGS patients) are unnecessarily exposed to steroids and their potential side effects^6^ and, at the same time, postponing alternative treatments that may have a better chance of success. What are needed are noninvasive diagnostic tests that can predict which patients are more likely to respond to steroids to better inform caregivers to make the appropriate clinical decisions. In this pilot study, we enrolled children diagnosed with idiopathic NS and compared the urine proteome of patients with SSNS to those with SRNS. We employed isobaric tags for relative and absolute quantitation (iTRAQ) labeling techniques for relative quantitation and identification of differentially expressed proteins. These methods, which originated from the isotope-coded affinity tag approach reported by Gygi et al,^7^ have the added advantages of using labeling chemistry targeted at primary amines (rather than sulfhydryl groups) and the ability to simultaneously measure relative quantities of proteins under multiple conditions.^8^ Differentially expressed proteins were validated using clinically available tools such as enzyme-linked immunosorbent assay (ELISA) and clinical immunonephelometry.

## Methods and Materials

### Patients and study design

Under an Institutional Review Board–approved protocol (2013-1715), informed consent was recorded from all participants and/or their legal guardians. Exclusion criteria included history of gross hematuria, active or recurrent urinary tract infection, or NS secondary to systemic disease. The study was performed in accordance with the principles of the Declaration of Helsinki. Urine and clinical data were collected from 50 patients, aged 2 to 19, who were diagnosed with idiopathic NS at Cincinnati Children’s Hospital Medical Center. The samples were collected over a period of 24 months. The study included 20 patients with SRNS (19 of whom had biopsy-proven FSGS) and 30 patients with SSNS. Urine was collected as part of a standard clinical visit, centrifuged at 5000 *g* for 5 minutes, aliquoted, and stored at −80°C. No more than 2 freeze-thaw cycles were used per sample. For our measurements, each patient is represented by a single sample. Demographic and clinical data, including urinalysis, steroid-response history, most recent serum creatinine, and current remission/relapse status, were recorded at the time of patient enrollment. Estimated glomerular filtration rate was calculated from serum creatinine using the new Schwartz formula^9^ and classified to chronic kidney disease (CKD) stage.^10^ Steroid-sensitive nephrotic syndrome was defined as the ability to reach remission within 8 weeks after initial diagnosis in response to steroid treatment, as evidenced by normalization of protein urine reading to a negative reading on a urine dipstick. Steroid-resistant nephrotic syndrome was defined as a failure to respond to standard steroid treatment (2 mg/kg/day) for at least 8 weeks.

### Quantitative profiling of urine proteins using isobaric protein labeling and tandem mass spectrometry

Urine samples from 2 subject groups (5 each from SRNS relapse and SSNS relapse) were prepared for quantitative protein profiling using the iTRAQ method^8^ by following the vendor instructions (Sciex, Toronto, ON, Canada). The patients selected for the 5 × 5 comparison met the following criteria—(1) all patients presented to the clinic with active disease consisting of high-grade proteinuria and (2) had samples collected within 6 months prior to the experiment to ensure the most pristine samples possible. The sample preparation protocol prior to iTRAQ tagging varied from the original vendor protocol; thus, the workflow is summarized here with details of each step provided below. The general sample preparation and analysis workflow included concentration and buffer exchange of each urine sample followed by preparative separation of the proteins on a mini sodium dodecyl sulfate polyacrylamide gel electrophoresis gel, in-gel trypsin digestion and recovery of the peptides, iTRAQ tagging of duplicate SSNS and SRNS samples with the iTRAQ 4-plex reagents (114, 115, 116, 117 reporters), combining the peptide from the 4 samples in equal portions, then subjecting peptides to nanoscale liquid chromatography coupled to tandem mass spectrometry (nanoLC-MS/MS), followed by protein identification and quantitation of the collective data set using the ProteinPilot (PP), ProteinPilot Descriptive Statistics Template (PDST), and Protein Alignment software algorithms (AB Sciex, Toronto, ON, Canada). Additional details of each step in the process are provided below.

#### Gel electrophoresis and isolation of peptides

Proteins from SRNS and SSNS urine samples were concentrated, and buffer exchanged (2×) with Invitrogen 1× Laemmli buffer using 3 kDa Amicon concentrator cartridge (UFC500396). The protein concentration for each sample was determined using the noninterfering (Ni) protein assay reagents from G-Biosciences (Maryland Heights, MO); 50 µg each from the 5 SRNS and 5 SSNS (10 samples total) were loaded onto separate lanes of a 1-dimensional (1D), 4% to 12% Bis-Tris minigel, then electrophoresed for 15 minutes which was just long enough for the proteins to enter into the gel. The gel region containing the proteins (about 1.5 cm × 2.5 cm) was cut from the gel and subjected to in-gel trypsin digestion and subsequent recovery of peptides as described previously.^11^

##### iTRAQ labeling

The isolated peptides from the 10 urine samples (5 SRNS and 5 SSNS) were each divided in half such that technical replicates were available for each sample. The patient samples were paired randomly between groups; 5 pairwise comparative groups (A-E) were tagged using the 4-plex iTRAQ reagents as described previously.^8^ The 114 and 115 reporter tags were used for the technical replicates of SRNS samples, whereas the 116 and 117 reporter tags were used for the technical replicates of the SSNS samples. After labeling, the samples were mixed together in equal quantities for subsequent separation, identification, and quantitative analysis.

#### Nanoliquid chromatography coupled to electrospray tandem mass spectrometry

Nanoliquid chromatography coupled to electrospray tandem mass spectrometry analyses were performed on a TripleTOF 5600+ (Sciex) attached to an Eksigent (Dublin, CA) nanoLC-ultra nanoflow system; 2.5 µg of total protein from each 4-plex mixture was loaded (via an Eksigent NanoLC-AS-2 autosampler) onto an IntegraFrit Trap Column (outer diameter of 360 µm, inner diameter of 100 µm, and 25 µm packed bed) from New Objective, Inc. (Woburn, MA) at 2 µL/min in formic acid/H2O 0.1/99.9 (v/v) for 15 minutes to desalt and concentrate the samples. For the chromatographic separation of peptides, the trap column was switched to align with the analytical column, Acclaim PepMap100 (inner diameter of 75 µm, length of 15 cm, C18 particle sizes of 3 µm, and pore sizes of 100 Å) from Dionex-Thermo Fisher Scientific (Sunnyvale, CA). The peptides were eluted using a variable mobile phase (MP) gradient from 95% phase A (formic acid/H2O 0.1/99.9, v/v) to 40% phase B (formic acid/acetonitrile 0.1/99.9, v/v) for 70 minutes, from 40% phase B to 85% phase B for 5 minutes, and then keeping the same MP composition for 5 more minutes at 300 nL/min. The nLC effluent was ionized and sprayed into the mass spectrometer using NANOSpray III Source (AB Sciex). Ion source gas 1 (GS1), ion source gas 2 (GS2), and curtain gas (CUR) were, respectively, kept at 7, 0, and 25 vendor specified arbitrary units. Interface heater temperature and ion spray voltage were kept at 150 C and at 2.3 kV, respectively. Mass spectrometer method was operated in positive ion mode set to go through 4156 cycles for 90 minutes, where each cycle performed 1 time of flight mass spectrometry scan type (0.25 seconds accumulation time, in a 400-1600 m/z window) followed by 20 information dependent acquisition mode MS/MS-scans on the most intense candidate ions having a minimum 150 counts. Each product ion scan was operated under vender specified high-sensitivity mode with an accumulation time of 0.05 seconds and a mass tolerance of 50 mDa. Former MS/MS-analyzed candidate ions were excluded for 10 seconds after its first occurrence, and data were recorded using Analyst-TF (v.1.6) software.

#### Data analyses of quantitative protein profiling

Individual and merged search from the nano-scale liquid chromatographic tandem mass spectrometry analyses was accomplished using PP software (version 4.5, revision 1656) that uses Paragon algorithm, against a SWISS-PROT database of human protein sequences. A vendor sample type including all biological modifications was selected for the search parameter as variable modification while methylthiocysteine was used as a fixed modification. The output files for the PP database search (*.group file) contain the peptide identification tables, protein identification tables, and relative quantitation data from the iTRAQ reporter ions from each peptide all of which can be exported as Excel spreadsheets for further statistical analysis using the PDST (version 3.005pB). The PDST is a mathematical Excel template that processes the relative quantitation data among the sample sets and provides statistical probabilities related to the confidence of the protein identification in relationship to an inverse (decoy) protein database, and provides *P* values regarding the relative quantitation of the 4 reporter ions for each protein. For protein identification and quantitative profiling, a minimum of 2 peptides at 99% or greater confidence was required. After confident protein profiles were collected for each of the 5 pairwise comparisons of the SRNS and the SSNS samples, the collective proteins from across all 5 groups were analyzed using the vendor supplied (Sciex) Protein Alignment Template algorithm (version 2.000p). This algorithm allows for the comparison of up to 10 pairwise groups to determine common protein changes across all the groups. The data reported here required that the proteins be detected in a minimum of 3 of the 5 samples and maintained statistical significance of *P* < .05 based on a *t*-test versus the null values.

### Urine measurements

Urine vitamin D–binding protein (VDBP) was measured using an ELISA kit that is available commercially (R&D Systems, Minneapolis, MN). Test variability (coefficients of variation [CVs]) were 5.9% (intra-assay) and 6.2% (interassay). The urine neutrophil gelatinase–associated lipocalin (NGAL) ELISA was performed using a commercially available assay (NGAL ELISA Kit 036; Bioporto, Grusbakken, Denmark) that specifically detects human NGAL. The intra-assay CV was 2.1% and interassay CV was 9.1%. Alpha-1 acid glycoprotein 2 (AGP2 or orosomucoid 2) was measured using a commercially available ELISA (Abnova—Taipei City, Taiwan) with an intra-assay CV of 4.4% and an interassay CV of 7.2%. Human fetuin-A and alpha-1 acid glycoprotein 1 (AGP1 or orosomucoid) were measured using commercially available ELISAs with CVs (intra/inter) of 5.5%/7.6% and 5.6%/7.2%, respectively. Human thyroxine-binding globulin (TBG) was measured using a commercially available ELISA (Kamiya Biomedical, Seattle, WA). Thyroxine-binding globulin had CVs of 8.2% (intra) and 10.1% (inter). Hemopexin and prealbumin (transthyretin) were measured with commercially available ELISAs (Assaypro, St. Charles, MO) with CVs of (intra/inter) of 4.9%/7.3% and 4.6%/9.0%, respectively. Alpha-2 macroglobulin was measured using immunonephelometry on a Siemens BNII clinical nephelometer (Siemens, Munich, Germany). Alpha-1-B glycoprotein (A1BG) was measured using a lab constructed ELISA as described previously.^12^

#### Statistical analysis on selected biomarkers measured at the patient level

Ten biomarkers selected after Quantitative Protein Profiling were further measured at the patient level using a total of 50 patients, 20 with SRNS and 30 with SSNS. Due to small sample numbers, and to add a level of verification to the proteomics methods, the samples from the 5 × 5 comparison were included in this analysis. As all the biomarkers showed right skewness of their empirical distributions, log_2_ transformations were used to correct the skewness and ensure that parametric statistical models could be used in analyses. Means with original values were presented after taking inverse function of the transformed means (ie, 2^transformed mean^) estimated from the statistical models. Two steps of statistical analyses were used in the study. In step 1 of analysis of association, each biomarker was compared of its means between SRNS and SSNS groups using 2 sample tests. In step 2 of predictive analysis, multivariate logistical regression models were used to predict SRNS using a panel of biomarkers. Here, we considered 2 candidate panels, one that employed all 10 biomarkers as the panel (or the predictors) in the multivariate logistical model (MLM-10), and the other that chose 5 biomarkers that showed significance in step 1 (MLM-5). The multivariate logistical regression model from each panel would calculate a logit or risk score of SRNS, and the score was evaluated for discriminative or diagnostic accuracy of SRNS using a receiver operating characteristic (ROC) curve. In particular, the overall accuracy could be evaluated using the area under the ROC curve (or AUC), and specific accuracy under a cutoff score could be evaluated using corresponding sensitivity and specificity. The accuracy is considered “outstanding,” “excellent,” “very good,” “fair,” and “poor” if an AUC is “0.9 to 1,” “0.8 to 0.89,” “0.7 to 0.79,” “0.6 to 0.69,” and “<0.6,” respectively, and a sensitivity or specificity is “0.8 to 1,” “0.6 to 0.79,” “0.4 to 0.59,” “0.2 to 0.39,” and “<0.2,” respectively. The comparison between an ROC curve from a multivariate model vs an ROC curve from an individual biomarker was tested using a nonparametric test.^13^ The same analyses were repeated in a subset of relapsed patients only. Subanalyses on relapsed patients were not performed given too small the sample size, especially those with SRNS (n = 3). All statistical analyses were performed using SAS 9.4 software (SAS, Cary, NC). *P* values <.05 were considered statistically significant.

## Results

### Patients

Fifty pediatric patients were enrolled over a 2-year period. Out of the 50 subjects, 20 presented with SRNS and 16 with FSGS on biopsy. Seventeen subjects had active disease and 3 were in remission. Thirty patients demonstrated response to corticosteroid therapy and were labeled SSNS at the time of sample collection, 14 SSNS patients had active proteinuria, and 16 presented without protein in their urine. Seventeen SRNS patients and the active SSNS patients had high-grade proteinuria as diagnosed by urine dipstick at the time of collection. The high-grade test result indicates protein levels greater than 2000 mg/dL. Patient demographics can be seen in Table 1. Steroid-resistant nephrotic syndrome patients differed from SSNS in age (12.3 vs 7.5 years, *P* < .001), ±hypertension (75% vs 30%, respectively, *P* = .003), pathologic diagnosis (FSGS vs no biopsy, *P* < .001), and current steroid therapy (SRNS 45% vs SSNS 87%, *P* = .001).

**Table 1.** Patient Demographics.

Variable	SRNS (n = 20)	SSNS (n = 30)	*P* value
Age, years; mean ± SE	12.3 ± 1.2	7.5 ± 0.8	.001
Sex, %, male	14 (70)	20 (68)	NS
**Pathology, %**			
FSGS	16 (80)	2 (6.7)	.001
MCD	1 (5)	7 (23.3)	
Other	2 (10)	0	
No biopsy	1 (5)	21 (70)	
Hypertension	15 (75)	9 (30)	.003
**Immunosuppressant, %**			
Steroid	9 (45)	26 (87)	.001
CNI	4 (20)	5 (17)	
MMF	3 (15)	1 (3)	
Rituximab	2 (10)	4 (13)	
CTX	2 (10)	3 (10)	
ACEI/ARB	8 (40)	1 (3)	
GFR, mL/min/1.73 m^2^	119 ± 11.4	135 ± 6.1	NS
MALB/Cr, mg/mg; ± SE	2.0 ± 0.6	1.5 ± 0.34	NS

Abbreviations: FSGS, focal segmental glomerulosclerosis; GFR, glomerular filtration rate; MCD, minimal change disease; SRNS, steroid-resistant nephrotic syndrome; SSNS, steroid-sensitive nephrotic syndrome; CNI, calcineurin inhibitor; MMF, mycophenolate mofetil; CTX, cytoxan; ACEI, angiotensin converting enzyme inhibitor; ARB, angiotensin receptor blocker; MALB, microalbumin; Cr, creatinine.

### iTRAQ profiling for differential proteins in SRNS vs SSNS

Samples from a cohort of 10 patients (5 in each group) were prepared in duplicate (see Table 2) using a 4-plex isotope tagging method (iTRAQ) followed by nanoLC-MS/MS profiling of the sample groups for protein identification and evaluation of quantitative changes as described in the “Materials and Methods” section. Collectively, more than 150 proteins were identified from the sample sets. Of these 150+ proteins identified, 72 proteins were identified and quantified in at least 3 of the 5 pairwise groups (see supplementary Table S1 for complete ratio values for all pairwise comparisons). Importantly, statistical analysis of the protein changes among the patient cohort revealed 13 protein changes with *P* values <.05. (Table 3). These 13 proteins were selected for further validation in a larger sample group.

**Table 2.** iTRAQ pairwise groupings.

4-plex group	Sample ID	Tag	Sample group
A	SRNS, sample 007, rep 1	114	Resistant
	SRNS, sample 007, rep 2	115	
	SSNS, sample 002, rep 1	116	Sensitive
	SSNS, sample 002, rep 2	117	
B	SRNS, sample 008, rep 1	114	Resistant
	SRNS, sample 008, rep 2	115	
	SSNS, sample 015, rep 1	116	Sensitive
	SSNS, sample 015, rep 2	117	
C	SRNS, sample 009, rep 1	114	Resistant
	SRNS, sample 009, rep 2	115	
	SSNS, sample 027, rep 1	116	Sensitive
	SSNS, sample 027, rep 2	117	
D	SRNS, sample 012, rep 1	114	Resistant
	SRNS, sample 012, rep 2	115	
	SSNS, sample 021, rep 1	116	Sensitive
	SSNS, sample 021, rep 2	117	
E	SRNS, sample 004, rep 1	114	Resistant
	SRNS, sample 004, rep 2	115	
	SSNS, sample 013, rep 1	116	Sensitive
	SSNS, sample 013, rep 2	117	

Abbreviations: iTRAQ, isobaric tags for relative and absolute quantitation; SRNS, steroid-resistant nephrotic syndrome; SSNS, steroid-sensitive nephrotic syndrome.

**Table 3.** Differential Protein Findings by iTRAQ.

Accession	Protein name	SSNS/SRNS Group A	SSNS/SRNS Group B	SSNS/SRNS Group C	SSNS/SRNS Group D	SSNS/SRNS Group E	Average log_2_	*P* value
sp|P02774|VTDB_HUMAN	VDBP	−0.668	−0.529	−0.628	−0.078	−0.919	−0.564	.015
sp|P02765|FETUA_HUMAN	Fetuin-A	−0.530	−0.466	−0.365		−0.278	−0.410	.005
sp|P02790|HEMO_HUMAN	Hemopexin	−0.328	−0.366	−0.337		−0.554	−0.396	.005
sp|P02766|TTHY_HUMAN	Prealbumin	−0.281	−0.531	−0.326	−0.052	−0.491	−0.336	.017
sp|P02647|APOA1_HUMAN	Apolipoprotein A-1	−0.139	−0.368	−0.332	−0.186	−0.575	−0.320	.014
sp|P01019|ANGT_HUMAN	Angiotensinogen	−0.263	−0.225	−0.260		−0.376	−0.281	.003
sp|P01024|CO3_HUMAN	Complement C3	−0.323	−0.098	−0.208	−0.059	−0.211	−0.180	.018
sp|P01023|A2MG_HUMAN	Alpha-2 macroglobulin	−0.139	−0.175		−0.172		−0.162	.005
sp|P02763|A1AG1_HUMAN	AGP1	0.140	0.177	0.183	0.101	0.086	0.138	.002
sp|P05543|THGB_HUMAN	TBG	0.126	0.240	0.349	0.213	0.056	0.197	.017
sp|P19652|A1AG2_HUMAN	AGP2	0.238	0.066	0.317	0.459	0.247	0.265	.014
sp|P25311|ZA2G_HUMAN	Zinc-alpha-2 glycoprotein	0.205	−0.013	0.529	0.437	0.366	0.305	.033
sp|P04217|A1BG_HUMAN	Alpha-1-B glycoprotein	0.120	0.324	0.733	0.681	0.173	0.406	.033

Abbreviations: AGPI, alpha-1 acid glycoprotein 1; AGP2, alpha-1 acid glycoprotein 2; SRNS, steroid-resistant nephrotic syndrome; SSNS, steroid-sensitive nephrotic syndrome; TBG, thyroxine-binding globulin; VDBP, vitamin D–binding protein.

### Validation

Of the 13 proteins determined to be different between the 2 groups, we were able to find reliable assays for 9 proteins. These proteins were included for validation using ELISA or immunonephelometry in the expanded cohort (N = 50): AGP, AGP2, alpha-1 microglobulin, A1BG, fetuin-A, hemopexin, prealbumin (transthyretin), TBG, and VDBP. In addition, we measured NGAL because we have previously shown to be able to differentiate SSNS from SRNS.^14^ Table 4 shows that VDBP (*P* < .001), prealbumin (*P* < .001), NGAL (*P* = .001), fetuin-A (*P* < .001), and AGP2 (*P* = .03) were all 5.5 to 38 fold higher in SRNS patients than SSNS in the complete cohort.

**Table 4.** Summary of biomarkers by SSNS/SRNS.

VARIABLE	Mean (95% CI)		Fold (SRNS/SSNS)	*P*
	SRNS	SSNS		
**All (N = 50)**				
	n = 20	n = 30		
*VDBP*	2519.41 (669.59–9479.56)	66.25 (22.46–195.47)	38.0	<.001
*NGAL*	30.77 (15.01–63.08)	5.57 (3.10–10.00)	5.5	.001
*Fetuin-A*	36 723.78 (13 878.94–97 171.38)	3433.82 (1551.44–7600.15)	10.7	<.001
*Prealbumin*	20 685.39 (7391.11–57 891.95)	1649.83 (712.04–3822.76)	12.5	<.001
*AGP2*	141.30 (54.38–367.14)	35.79 (16.41–78.04)	3.9	.030
AGP1	90.97 (13.43–616.16)	82.89 (17.38–395.22)	1.1	.940
A2MCG	119.93 (40.33–356.62)	35.79 (14.70–87.13)	3.4	.090
A1BG	310.97 (146.86–658.43)	192.57 (104.37–355.31)	1.6	.325
TBG	1136.19 (320.34–4029.90)	730.91 (259.97–2054.98)	1.6	.590
Hemopexin	4701.67 (1993.48–11 089.00)	2049.40 (1017.11–4129.39)	2.3	.138
**Relapse (N = 31)**				
	n = 17	n = 14		
*VDBP*	3708.40 (1010.16–13 613.90)	353.58 (84.36–1482.06)	10.5	.018
*NGAL*	33.48 (15.22–73.64)	7.16 (3.00–17.06)	4.7	.011
*Fetuin-A*	55 745.38 (23 435.74–132 598.64)	15 607.72 (6006.81–40 554.13)	3.6	.053
*Prealbumin*	33 079.70 (12 129.94–90 212.00)	5000.48 (1655.35–15 105.43)	6.6	.014
*AGP2*	171.01 (81.37–359.43)	266.72 (117.65–604.69)	0.6	.422
AGP1	141.97 (22.88–881.03)	1340.72 (179.35–10 022.32)	0.1	.103
A2MCG	137.11 (44.26–424.79)	110.19 (31.70–383.10)	1.2	.795
A1BG	318.05 (139.00–727.74)	241.52 (97.01–601.29)	1.3	.655
TBG	1639.78 (419.97–6402.53)	1237.83 (275.92–5553.08)	1.3	.781
Hemopexin	4019.45 (1583.99–10 199.55)	3126.86 (1120.64–8724.72)	1.3	.717

Abbreviations: AGPI, alpha-1 acid glycoprotein 1; AGP2, alpha-1 acid glycoprotein 2; CI, confidence interval; NGAL, neutrophil gelatinase–associated lipocalin; SRNS, steroid-resistant nephrotic syndrome; SSNS, steroid-sensitive nephrotic syndrome; VDBP, vitamin D–binding protein. Italicized font represents biomarker included in MLM-5.

**Figure 1.**
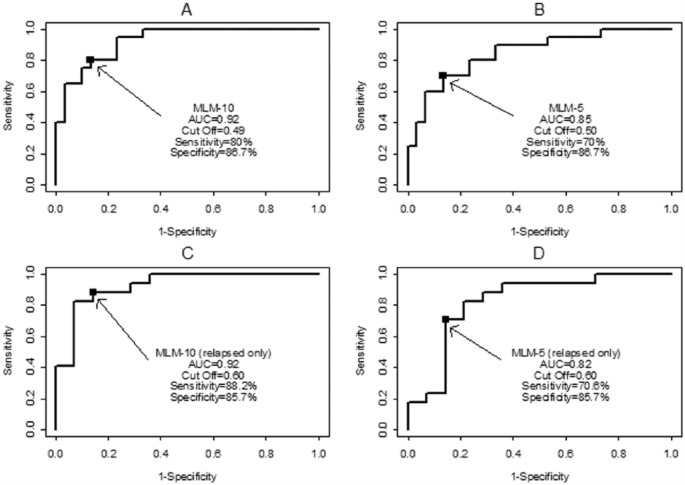
ROC curves using panels of 10 biomarkers (MLM-10) and 5 biomarkers (MLM-5), respectively. A) MLM-10, all patients; B) MLM-5, all patients; C) MLM-10, relapse only; D) MLM-5 relapse only. ROC indicates receiver operating characteristic. Arrows indicate Cut Off values.

The predictive analyses showed the panel of biomarkers (MLM-5) improved the AUC to 0.85, significantly higher than that of AGP2 or any individual biomarker not selected in the panel. The panel using all 10 biomarkers (MLM-10) yielded an AUC of 0.92, significantly higher than that of any single biomarker (Table 5). Sensitivities and specificities from panels showed excellent outstanding accuracy under suggested cutoff scores (see Table 6 and Figure 1). Table 7 provides the algorithms to calculate the risk scores of SRNS of the panels. For the algorithms, the formula in step 2 is derived from the logistic regression analysis, and the cutoff for step 6 was derived from the ROC curve analysis. To better understand these algorithms, an example is provided to demonstrate the steps to calculate the risk score and draw a conclusion of Steroid Resistant (or Steroid Sensitive), using a predefined cut. The example is based on Model MLM-5; similar calculations apply to other models in the table.

**Table 5.** Summary of AUC for detecting SSNS.

ROC model	AUC (95% CI)	*P* vs MLM_10	*P* vs MLM_5
**All (N = 50)**			
MLM-10	0.92 (0.85–0.99)	—	.076
MLM-5	0.85 (0.74–**0.96)**	.076	—
* VDBP*	0.81 (0.68–**0.95)**	.052	.267
* NGAL*	0.78 (0.65–**0.91)**	.020	.264
* Fetuin-A*	0.78 (0.65–**0.91)**	.016	.195
* Prealbumin*	0.78 (0.65–**0.91)**	.026	.286
* AGP2*	0.65 (0.49–**0.80)**	.001	.011
AGP1	0.55 (0.39–0.71)	.000	.000
A2MCG	0.64 (0.48–0.80)	.001	.027
A1BG	0.59 (0.42–0.75)	.000	.008
TBG	0.56 (0.39–0.73)	.000	.003
Hemopexin	0.66 (0.50–0.82)	.002	.028
**Relapse (N = 31)**			
MLM-10	0.92 (0.83–1.00)	—	.129
MLM-5	0.82 (0.66–0.99)	.129	—
* VDBP*	0.77 (0.58–0.96)	.105	.561
* NGAL*	0.76 (0.58–0.94)	.037	.312
* Fetuin-A*	0.68 (0.48–0.88)	.016	.118
* Prealbumin*	0.73 (0.55–0.91)	.035	.215
* AGP2*	0.60 (0.39–0.80)	.003	.067
AGP1	0.57 (0.35–0.79)	.002	.091
A2MCG	0.52 (0.30–0.73)	.001	.023
A1BG	0.58 (0.36–0.79)	.005	.051
TBG	0.57 (0.36–0.78)	.003	.045
Hemopexin	0.56 (0.35–0.77)	.003	0.080

Abbreviations: AGPI, alpha-1 acid glycoprotein 1; AGP2, alpha-1 acid glycoprotein 2; AUC, area under the curve; CI, confidence interval; NGAL, neutrophil gelatinase–associated lipocalin; ROC receiver operating characteristic; SSNS, steroid-sensitive nephrotic syndrome; TBG, thyroxine-binding globulin; VDBP, vitamin D–binding protein. Italicized font indicates a biomarker included in the MLM-5.

**Table 6.** Sensitivity and specificity of detecting SSNS using suggested cutoffs from multivariate logistic models.

Model	AUC (95% CI)	Cutoff probability	Sensitivity	Specificity
**All (N = 50)**				
MLM-10	0.92 (0.85–0.99)	.49	80.0%	86.7%
MLM-5	0.85 (0.74–0.96)	.50	70.0%	86.7%
**Relapse (N = 31)**				
MLM-10	0.92 (0.83–1.00)	.60	88.2%	85.7%
MLM-5	0.82 (0.66–0.99)	.60	70.6%	85.7%

Abbreviations: AUC, area under the receiver operating characteristic curve; SSNS, steroid-sensitive nephrotic syndrome.

**Table 7.** Algorithms of computing risk scores of SRNS.

Step	MLM-10 (any patient)	MLM-5 (any patient)	MLM-10 (relapsed patient only)	MLM-5 (relapsed patient only)
1	Converting all biomarkers into log_2_ values	Converting all biomarkers into log_2_ values	Converting all biomarkers into log_2_ values	Converting all biomarkers into log_2_ values
2	Each biomarker is adjusted by a multiplier in the following: 0.27 × VDBP −0.004 × Prealbumin 0.51 × NGAL 0.37 × fetuin-A 0.50 × AGP2 −0.49 × AGP1 0.22 × A2MCG 0.20 × Hemopexin −0.09 × TBG 0.11 × A1BG	Each biomarker is adjusted by a multiplier in the following: 0.23 × VDBP −0.03 × Prealbumin 0.32 × NGAL 0.01 × fetuin-A 0.03 × AGP2	Each biomarker is adjusted by a multiplier in the following: 0.23 × VDBP 0.07 × prealbumin 0.82 × NGAL 0.41 × fetuin-A 0.42 × AGP2 −0.60 × AGP1 0.24 × A2MCG 0.36 × Hemopexin −0.15 × TBG 0.24 × A1BG	Each biomarker is adjusted by a multiplier in the following: 0.14 × VDBP 0.16 × prealbumin 0.26 × NGAL −0.05 × fetuin-A −0.47 × AGP2
3	Sum of the adjusted biomarkers	Sum of the adjusted biomarkers	Sum of the adjusted biomarkers	Sum of the adjusted biomarkers
4	Calculate a raw score by subtracting 13.65 from the sum in step 3	Calculate a raw score by subtracting 3.58 from the sum in step 3	Calculate a raw score by subtracting 17.27 from the sum in step 3	Calculate a raw score by subtracting 0.03 from the sum in step 3
5	Calculate the risk score by taking 2 to the power of the raw score in step 4	Calculate the risk score by taking 2 to the power of the raw score in step 4	Calculate the risk score by taking 2 to the power of the raw score in step 4	Calculate the risk score by taking 2 to the power of the raw score in step 4
6	Compare the risk score with the cutoff point 0.49: SRNS positive if the score > cutoff SRNS negative if the score ⩽ cutoff	Compare the risk score with the cutoff point 0.50: SRNS positive if the score > cutoff SRNS negative if the score ⩽ cutoff	Compare the risk score with the cutoff point 0.60: SRNS positive if the score > cutoff SRNS negative if the score ⩽ cutoff	Compare the risk score with the cutoff point 0.60: SRNS positive if the score > cutoff SRNS negative if the score ⩽ cutoff

Abbreviations: AGPI, alpha-1 acid glycoprotein 1; AGP2, alpha-1 acid glycoprotein 2; NGAL, neutrophil gelatinase–associated lipocalin; SRNS, steroid-resistant nephrotic syndrome; TBG, thyroxine-binding globulin; VDBP, vitamin D–binding protein.

Assume a patient’s observed values of VDBP, prealbumin, NGAL, fetuin-A, and AGP2 are 250, 900, 24, 1250, and 50, respectively:

Step1: The log_2_ transferred values are 7.97, 9.81, 4.58, 10.29, and 5.64, respectively.
Step2: The formula is 0.23 × 7.97 − 0.03 × 9.81 + 0.32 × 4.58 + 0.01 × 10.29 + 0.03 × 5.64.
Step3: The sum of step 2 is 3.2765.
Step4: The adjusted sum is 3.2765 − 3.58 = −0.3035.
Step5: The risk score = 2 ^ 0.3035 = 1.23
Step6: As the risk score = 1.23 > 0.5, the patient is considered likely Steroid Resistant based on this model.

Similar conclusions could be reached in the subanalyses on relapsed patients.

## Discussion

Patients with SRNS have a more progressive disease course and related poor outcomes when compared patients with SSNS.^15-18^ The number of cases of FSGS and the resulting SRNS in the pediatric population are continuing to increase.^19-21^ Currently, the only method of diagnosis is an invasive biopsy, which is not typically performed in children until first-line treatments fail. This results in patients with SRNS getting an unnecessary exposure to high-dose corticosteroids and a delay in initiating a more appropriate treatment. In this study, our objective was to use a robust proteomic technique, iTRAQ, to identify potential biomarkers that could be used to noninvasively distinguish steroid-resistant patients from those whose disease is likely to respond to steroids. Of the 13 differentially expressed proteins identified by iTRAQ, we were able to use ELISA and immunonephelometry to validate a 10-biomarker panel with an excellent discriminatory power to identify SRNS (AUC, 0.92) in both the complete cohort and the subset with active disease. In addition, we demonstrated that by using the 5 markers with significant association to SRNS, we were still able to achieve an AUC of 0.85 in the complete cohort and an AUC of 0.82 in the active disease subjects. This predictive biomarker panel includes VDBP, NGAL, fetuin-A, prealbumin, and AGP2.

Our study is the first to use the iTRAQ profiling to find potential biomarkers to distinguish SRNS from SSNS and validate the findings in a pediatric population. Therefore, it is not surprising that we found markers that differ from earlier studies. For instance, in one such study, Khurana et al^22^ found that beta-2 microglobulin was associated with SRNS, but a separate validation was never undertaken. Those studies had used techniques, such as surface-enhanced laser desorption ionization time of flight mass spectrometry, that have fallen out of favor due to the fact that they only identify peptide or protein peaks—and one has to separately isolate the protein of the appropriate size and subsequently identify the protein using tandem MS.

The current study adds further validation to the previous findings concerning VDBP and NGAL in SRNS. Vitamin D–binding protein and NGAL have previously been shown to be increased in children with SRNS.^14,23^ While both had been shown previously to be correlated with proteinuria,^24,25^ their ability to positively identify patients with SRNS vs SSNS was independent of proteinuria as measured by MALB/Cr.^23^ It was found that VDBP was independently able to categorize (AUC, 0.87, *P* < .0002) SRNS and SSNS patients, without relation to proteinuria. This indicates a potential mechanism that leads to increased urine vitamin D binding protein (uVDBP) specifically in patients with SRNS. A plausible mechanism may involve intact megalin and cubulin receptors in the proximal tubule to reabsorb filtered VDBP. As SRNS leads to chronic tubular injury, this could explain increased levels of uVDBP in the urine. Supporting this theory, VDBP was recently shown in a rat model of adriamycin-induced nephrosis to be a biomarker of tubular fibrosis and renal interstitial damage.^26^

Interestingly, fetuin-A has been shown to work through megalin-mediated endocytosis to counter nephrocalcification in the tubular lumen in rats.^27^ Therefore, like VDBP, increased excretion of fetuin-A in the urine could be explained by megalin dysfunction and could represent a mechanism for the appearance of these proteins at high levels in the urine of SRNS patients. It has been suggested by some that the normal glomerulus leaks proteins at a nephrotic level, but that those proteins are generally reabsorbed in the proximal tubule by megalin and related proteins.^28^ However, in the nephrotic kidney, levels of megalin, clathrin, and other important parts of the endocytic pathway are compromised, which leads to albuminuria. Given the number of podocyte mutations discovered in nephrotic diseases such as FSGS,^29^ it is unlikely that megalin dysfunction accounts for all aspects of the disease, but it remains an intriguing possibility given some of our findings. Research indicates that fetuin-A may well be a biomarker for other forms of renal disease. Inoue et al^30^ demonstrated that fetuin-A was a risk factor for both microalbuminuria and reduction of glomerular filtration rate in diabetic nephropathy and could therefore be used as a marker to predict progression of the disease. Urinary fetuin-A has also been shown to be a sensitive (94%), yet not especially specific (60%), marker for progression and prediction of renal insufficiency in autosomal dominant polycystic kidney disease. Fetuin-A, similar to NGAL, appears to be a sensitive marker of progression of disease but lacks some specificity to individual disease processes.^31,32^

Not all of the markers we discovered have a track record as being associated with CKD in the urine. For instance, serum prealbumin levels are often elevated in CKD and are used to evaluate nutritional status in dialysis patients,^33^ but urinary levels have not appeared to be associated with specific diseases in the literature.

This study has certain limitations that must be presented. Our pilot study was cross-sectional, at a single center, and included a small subject pool, many of which had begun treatment at enrollment. We are therefore limited in the conclusions we can make about the *predictive* value of the biomarker panel. There exists an inherent age difference between our SRNS patients and our SSNS patients, as well as varying lengths of disease duration. This age difference is unavoidable in a limited population of patients with NS because the different forms tend to occur at different stages of life. For instance, most children with MCD are diagnosed before the age of 5, whereas FSGS occurs mainly after the age of 6.^2^ The strength of our panel to distinguish SRNS from SSNS (AUC, 0.93; *P* < .0001) is high enough, however, that it is not likely to represent an artifact of age differences. It is also unfortunate that we did not have access to serum samples for this study, so we could not ascertain whether any of these markers represent high serum levels that are leaking into the urine. Clinically, in the pediatric NS population, our results indicate a level of promise that our biomarker panel could be used to identify patients with the steroid-resistant form of the disease. Of course, our panel is only a tool and, as with any other diagnostic test, must be used in the context of the clinical situation to help inform the decision making of the physician.^34^

This biomarker panel must now be subjected to a larger, multicenter, prospective study which would allow us to access adequate numbers of patients with primary NS before initial treatments are administered and allow more control over age variation. This type of study would allow us to determine the utility of our panel in the early diagnosis of NS, and to determine whether it could be used to guide treatment, thus limiting exposure to high-dose steroids and their harmful side effects in patients who are not likely to benefit from their use. We could also determine whether any of the biomarkers in our panel change with response to therapy. Biomarkers that can be used in clinical trials as surrogate endpoints can allow for expedited drug development. The development of a noninvasive panel of urinary biomarkers that may be useful in guiding therapy for patients with NS could lead to improved care and better outcomes for patients with this progressive and devastating disease.

## Supplementary Material

Supplementary material
